# Perceptions and Feelings of Brazilian Health Care Professionals Regarding the Effects of COVID-19: Cross-sectional Web-Based Survey

**DOI:** 10.2196/28088

**Published:** 2021-10-22

**Authors:** Roberta Pires Corrêa, Helena Carla Castro, Bruna Maria Castro Salomão Quaresma, Paulo Roberto Soares Stephens, Tania Cremonini Araujo-Jorge, Roberto Rodrigues Ferreira

**Affiliations:** 1 Program in Education in Biosciences and Health Oswaldo Cruz Institute Oswaldo Cruz Foundation (Fiocruz) Rio de Janeiro Brazil; 2 Sciences, Technology and Inclusion Federal Fluminense University Niterói Brazil; 3 Laboratory of Functional Genomics and Bioinformatics Oswaldo Cruz Institute Oswaldo Cruz Foundation (Fiocruz) Rio de Janeiro Brazil; 4 Laboratory of Innovations in Therapies, Education and Bioproducts Oswaldo Cruz Institute Oswaldo Cruz Foundation (Fiocruz) Rio de Janeiro Brazil

**Keywords:** COVID-19, SARS-CoV-2, health professionals, Brazil, pandemic, mental health, health planning

## Abstract

**Background:**

The importance of health professionals has been recognized in COVID-19 pandemic–affected countries, especially in those such as Brazil, which is one of the top 3 countries that have been affected in the world. However, the workers’ perception of the stress and the changes that the pandemic has caused in their lives vary according to the conditions offered by these affected countries, including salaries, individual protection equipment, and psychological support.

**Objective:**

The purpose of this study was to identify the perceptions of Brazilian health workers regarding the COVID-19 pandemic impact on their lives, including possible self-contamination and mental health.

**Methods:**

This cross-sectional web-based survey was conducted in Brazil by applying a 32-item questionnaire, including multiple-choice questions by using the Google Forms electronic assessment. This study was designed to capture spontaneous perceptions from health professionals. All questions were mandatory and divided into 2 blocks with different proposals: personal profile and COVID-19 pandemic impact.

**Results:**

We interviewed Brazilian health professionals from all 5 Brazilian regions (N=1376). Our study revealed that 1 in 5 (23%) complained about inadequate personal protective equipment, including face shields (234/1376, 17.0%), masks (206/1376, 14.9%), and laboratory coats (138/1376, 10.0%), whereas 1 in 4 health professionals did not have enough information to protect themselves from the coronavirus disease. These professionals had anxiety due to COVID-19 (604/1376, 43.9%), difficulties in sleep (593/1376, 43.1%), and concentrating on work (453/1376, 32.9%). Almost one-third experienced traumatic situations at work (385/1376, 28.0%), which may have led to negative feelings of *fear of COVID-19* and *sadness*. Despite this situation, there was *hope* and *empathy* among their positive feelings. The survey also showed that 1 in 5 acquired COVID-19 with the most classic and minor symptoms, including headache (274/315, 87.0%), body pain (231/315, 73.3%), tiredness (228/315, 72.4%), and loss of taste and smell (208/315, 66.0%). Some of their negative feelings were higher than those of noninfected professionals (fear of COVID-19, 243/315, 77.1% vs 509/1061, 48.0%; impotence, 142/315, 45.1% vs 297/1061, 28.0%; and fault, 38/315, 12.1% vs 567/1061, 53.4%, respectively). Another worrying outcome was that 61.3% (193/315) reported acquiring an infection while working at a health facility and as expected, most of the respondents felt affected (344/1376, 25.0%) or very affected (619/1376, 45.0%) by the COVID-19.

**Conclusions:**

In Brazil, the health professionals were exposed to a stressful situation and to the risk of self-contamination—conditions that can spell future psychological problems for these workers. Our survey findings showed that the psychological support for this group should be included in the future health planning of Brazil and of other hugely affected countries to assure a good mental health condition for the medical teams in the near future.

## Introduction

In December 2019, Chinese authorities notified the World Health Organization (WHO) of several cases of pneumonia of unknown etiology in the city of Wuhan [1]. In January 2020, a new coronavirus, the severe acute respiratory syndrome coronavirus 2 (SARS-CoV-2), was identified from a patient’s throat swab sample [2,3], and the WHO named the disease as “Coronavirus Disease 2019 (COVID-19)” [4,5]. On January 30, 2020, 7736 cases were confirmed in China while 82 confirmed cases were detected in 18 other countries [5,6]. On this same day, WHO declared the SARS-CoV-2 outbreak as a global health emergency [7].

The first case of COVID-19 in South America was described in Brazil in February 2020. It was a man returning from a trip to Italy, where a significant outbreak was ongoing [8]. Since then, the pandemic spread fast in Brazil, producing an emergency state. To control COVID-19, the Brazilian Ministry of Health recommended measures of social distancing, use of masks, and hand hygiene [9]. The disease spread in large capitals, followed by an increase of COVID-19 cases in smaller cities and poorer communities as well [10]. In the middle of the COVID-19 outbreak, Brazil was considered the second most affected country. On January 25, 2021, Brazil was considered as the third country with the highest number of COVID-19 cases worldwide (8.8 million) by WHO, behind India (10.6 million) and United States of America (24.7 million) [11].

Based on the report of the first 425 confirmed cases in Wuhan, the common symptoms detected included fever, dry cough, myalgia and fatigue, headache, hemoptysis, abdominal pain, and diarrhea [12]. Furthermore, studies have reported severe cases of COVID-19 with pneumonia, intestinal, liver, thrombotic, and neuronal diseases, acute respiratory distress, multiple organ failure, and death [13,14]. Because of the efforts of different countries and pharmaceutical industries, the production of more than 5 types of vaccines started and is being slowly distributed worldwide. Meanwhile, there are no specific therapies available for those already infected, who have access only to support medical assistance [15].

In the affected countries, including Brazil, a rapid increase in the demand for health services occurred, mainly for hospital beds in intensive care units [16]. The pandemic has severely affected the way of living of many people and has disrupted the already precarious health system in several countries [17]. The historic challenges regarding an insufficient number of health professionals [18] and the increase in confirmed cases led to overburdening of these individuals. COVID-19 changed not only the daily routine of business, schools, lifestyle, and economics but also profoundly changed routines inside hospitals, some of which now may not attend to diseases other than COVID-19 owing to its huge life-threatening risk [19].

Recently, researchers have described the afflictions experienced by people during the pandemic period [20,21] that goes from changing personal behavior to psychological distress, anxiety, depression, and stress. Following daily life changes, these studies have shown an important behavioral change at the beginning of the pandemic, also leading to fear of COVID-19 and insecurity. This whole process is a crucial reaction, which is mainly caused by inefficient measures to control the pandemic and the lack of psychological assistance.

During the pandemic, the world has faced shutdown, slowdown, or lockdown, and individuals have been encouraged to use masks and practice social distancing. Meanwhile, health professionals had to go in the opposite direction. These workers were directly involved in offering diagnosis and treatment care for SARS-CoV-2-infected patients with almost uninterrupted work in a life-threatening, and sometimes, frustrating perspective. Lately, besides the feasible SARS-CoV-2 contamination risk, these professionals are also at a high risk of developing psychological distress and other mental health symptoms [22]. Thus, health care professionals have been considered as one of the most vulnerable working categories to develop psychological stress and other mental health symptoms, especially in countries highly affected, such as Brazil, which now faces another wave of a new coronavirus mutant (N501Y), which is at least 50% more infective than the original strain [23]. In this work, our purpose was to identify the impact of the COVID-19 pandemic on the life and work routine of Brazilian health care professionals through the study of their self-declared perceptions and their needs during this period.

## Methods

### Survey Questionnaire and Validation

This study was a cross-sectional web-based survey conducted in Brazil. We prepared a 32-items questionnaire using the Google Forms electronic assessment. A combination of structured (yes/no), multiple-choice selections with 1 final open question was used. All questions were mandatory and divided into 2 blocks with different proposals: (1) personal profile (eg, age, gender, ethnicity, household income, schooling level, and professional characteristics) and (2) COVID-19 pandemic impact. Our objective in most questions was to reflect on the perceptions of health professionals about COVID-19 and allow the analysis based on the respondent’s declarations during the pandemic. It is important to note that any diagnosis pointed by the participants was not debated with them nor were they requested for any documents to assure the pathological situation or diagnosis. The questionnaire underwent an internal validation by both an expert panel of 5 and in a respondent set of 10 health professionals from a huge national health institution (Fiocruz). Experts critically reviewed the instrument and offered important feedback such as addition, deletion, and reformulation of questions and answers, and errors in the form systems used to create the questionnaire. The first approach of the survey asked for informed consent and for the autodeclaration status of “health professionals,” considering all the workers in any type of unit of the National Brazilian Health System (Sistema Único de Saúde).

### Recruitment and Sample

The invitation to answer the questionnaire was distributed nationally to health professionals in different health institutions through emails, WhatsApp groups, and social media (Facebook). This study was designed to capture spontaneous perceptions from health professionals and had no epidemiological purpose. Their motivation to access and answer the forms relied on the altruistic feeling of the participant to collaborate with the research. Although the survey was not designed to follow strict representative numbers of health professionals in all Brazilian regions, a study of the last available census of Brazilian health professionals [24] was previously prepared to ascertain that all the geographical regions were covered with a sufficient representativity to be considered a national assessment. To reduce the bias of the result at a specific point, the survey was kept open around 3 weeks, from September 12 to October 5, 2020, collecting 1476 answers in the closure of the investigation. The final set of data was obtained after excluding duplicate answers through email confirmation (n=88) and answers in which the participant presented a contradictory statement related to his/her status of the health professional (n=12, retired, I am not working yet, I am not in the health area, salesman, primary school teacher), achieving 1376 answers that were finally analyzed.

### Data Analysis

Data exploration, analysis, and cleaning were performed using the Python programming language (version 3.6) with the Jupyter interface. During the analysis, the percentage of participants who selected each response was computed, and the Pandas and NumPy libraries were used together with Matplotlib library for the table generation. Chi-square analysis was performed whenever necessary to statistically confirm differences between any specific group of interest. The participants indicated using a 5-point scale how much COVID-19 affected their lives (1=not affected and 5=very affected) and since the beginning of the pandemic, how much they thought about COVID-19 (1=not at all and 5=very much). The level of anxiety was measured by averaging the participants’ scores (ranging from 1 to 5) so that the higher the average, the greater the anxiety of the individual was expected regarding COVID-19. Word clouds were prepared in the WordArt program. This approach was previously validated by other studies [25].

### Ethical Committee Approval

The ethical approval for this research was obtained from the Research Ethics Committee of the Oswaldo Cruz Institute-CEP FIOCRUZ/IOC under the number CAAE: 34985420.0.0000.5248. All respondents gave informed consent before their entry into the study.

## Results

After the web-based questionnaire distribution, 1376 answers came from all 5 Brazilian regions in a regional percentage distribution, following the same trends observed in the data from the last census of the health care professionals available at the Brazilian Health Ministry. The analysis of the demographic part of the questionnaire section showed that most of the respondents were females (1159/1376, 84.2%) in the age range of 31-50 years (830/1376, 60.3%) (Table 1). The female proportion in the survey was higher in the general population, but in the health profession, this is common owing to the influence of nursing and auxiliary nursing staffs that are ~85% females [26]. Accordingly, we found that the nursing staff (graduate/postgraduate nurses, nursing technicians, and nursing auxiliary) was the largest group answering this survey (669/1376, 48.6%). Since health staffs have a wide variety of professionals—partly legally regulated and others dealing with new professions that are under legislation [27,28]—the survey proposed 10 professional categories but registered 33 types of professions. In the descending order, the survey registered answers from nursing technicians/auxiliary (447/1376, 32.5%), nurses (228/1376, 16.6%), medical doctors (129/1376, 9.3%), physiotherapists/physical educators (128/1376, 9.2%), laboratory, radiology, and other technicians and technologists (75/1376, 5.5%), pharmacy professionals (50/1376, 3.6%), health community agents (17/1376, 1.2%), dentists (14/1376, 1.0%), administrative staff (12/1376, 1.0%), other types of health agents (n=6), and other 13 types of professions including mental therapy workers, social assistants, speech therapists, nutrition professionals, biologists, biomedical scientists, and others (270/1376, 19.6%). The wide reach of our survey corresponds to the general profile of the health professionals produced by the Brazilian Health Ministry at 1 month before the study [28], thus confirming that the survey participants represent this category of workers for the study of their perceptions.

**Table 1:** Profile of the Brazilian health professionals enrolled in this study (N=1376).

Demographic characteristics			Values, n (%)
**Brazilian regions**			
	Southeast	929 (67.5)	
	South	149 (10.8)	
	Central West	140 (10.2)	
	Northeast	92 (6.7)	
	North	66 (4.8)	
**Gender**			
	Female	1159 (84.2)	
	Male	215 (15.6)	
	Nonidentified	2 (0.1)	
**Age (years)**			
	18-30	287 (20.9)	
	31-40	467 (33.9)	
	41-50	363 (26.4)	
	51-60	201 (14.6)	
	>60	58 (4.2)	
**Ethnicity**			
	European-derived	724 (53.6)	
	African-derived	601 (43.7)	
	Asiatic	23 (0.0)	
	Indigenous	2 (0.0)	
	Nonidentified	26 (0.0)	
**Educational level**			
	University grade/postgraduate	903 (65.6)	
	Complete technical/high school level	438 (31.8)	
	Incomplete university grade	30 (2.2)	
	Incomplete technical/high school level	5 (0.4)	
**Household monthly income (USD)**			
	<52 USD	10 (0.7)	
	>52-260 USD	33 (2.4)	
	>260-500 USD	403 (29.3)	
	>500-1500 USD	598 (43.5)	
	≥1500 USD	332 (24.1)	
**Sharing the house with family/friends**			
	No	44 (3.2)	
	1-3 persons	1033 (75.1)	
	≥4 persons	299 (21.7)	

More than 50% of the respondents declared themselves as European-derived people (724/1376, 53.6%), with African-derived people constituting 43.7% (601/1376) of the participants (Table 1)—a proportion lower than that in the general composition of the Brazilian population, formed in 2010 majorly by African-derived people (50.9%). Concerning the education level, 31.8% (438/1376) completed the technical level and 65.6% (903/1376) had university grades (Table 1), as expected for the health working force [29,30]. Approximately 67.6% (930/1376) of the respondents had a family income higher than 500 USD and lived with 1-3 persons at home (Table 1). Among them, 39.7% (546/1376) were frontline health care workers during the COVID-19 outbreak, and only 19% were not working due to unemployment, retirement, or temporary leave from work due to risk factors for COVID-19 infection (Table 2). We also analyzed the amount of distress in relation to economic income and educational qualification, but no correlation was identified between these factors in this group of participants. In this survey of health professionals, 55.0% (757/1376) of them worked in the public sector and 76.8% (1057/1376) of them reported that all personal protective equipment (PPE) was available (Table 2). According to this, 23.2% (319/1376) who complained about inadequate PPE said that the scarcest items were face shields (234/1376, 17.0%), masks (206/1376, 14.9%), and laboratory coats (138/1376, 10.0%). One in 4 health professionals who answered the survey reported that they had not enough information to protect themselves from the coronavirus disease (360/1376, 26.2%). Regarding their personal information source, 40.0% (551/1376) reported the data published by the Brazilian Ministry of Health or the WHO, 26.7% (368/1376) on television, and 18.5% (254/1376) on social networks (Facebook/Instagram/WhatsApp/internet).

When asked about being infected by SARS-CoV-2, almost 22.9% (315/1376) reported positiveness (Table 3), confirming recent data showing rates of infection from 17.8% to 25% depending on the specific type of health profession [28]. However, a major proportion of the respondents did not know if they got infected (289/1376, 21.0%) and 56% (771/1376) reported that did not get COVID-19 (Table 3). Those who were infected by SARS-CoV-2 (315/1376, 22.9%) described majorly (263/315, 83.5%) 3 or more symptoms, with only 5.4% (17/315) being asymptomatic. The most recurrent symptoms were headache (274/315, 87.0%), body pain (231/315, 73.3%), tiredness (228/315, 72.4%), and loss of taste and smell (208/315, 66.0%). Regarding the worst outcome and the severe form of COVID-19, 6.0% (19/315) of the health professionals responding to the survey reported experiences of hospitalization and 0.3% (1/315) reported receiving intubation and invasive ventilation in the intensive care units (Table 3).

Critically, 61.3% (193/315) answered that they were infected with SARS-CoV-2 while working at a health facility, whereas 15.2% (48/315) did not know where they were infected, and 13.3% (42/315) assumed that they got infected from their own family members that had COVID-19. An important result is that 48.3% (152/315) of those health professionals who acquired COVID-19 reported that family members or friends living in the same house also got infected, and 27.0% (85/315) think that they probably were the source of their infection (Table 3).

**Figure 1:**
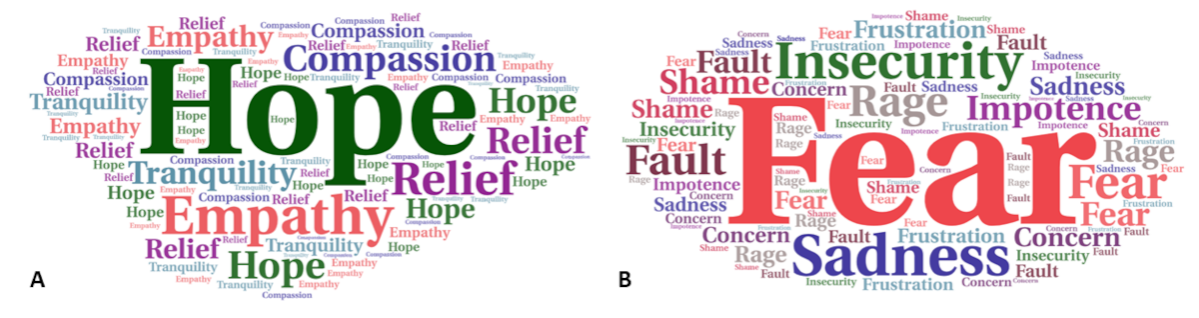
Word cloud images showing the qualitative frequencies of positive (A) and negative (B) feelings reported by health professionals in the COVID-19 perception survey conducted in Brazil (September-October 2020).

**Figure 2:**
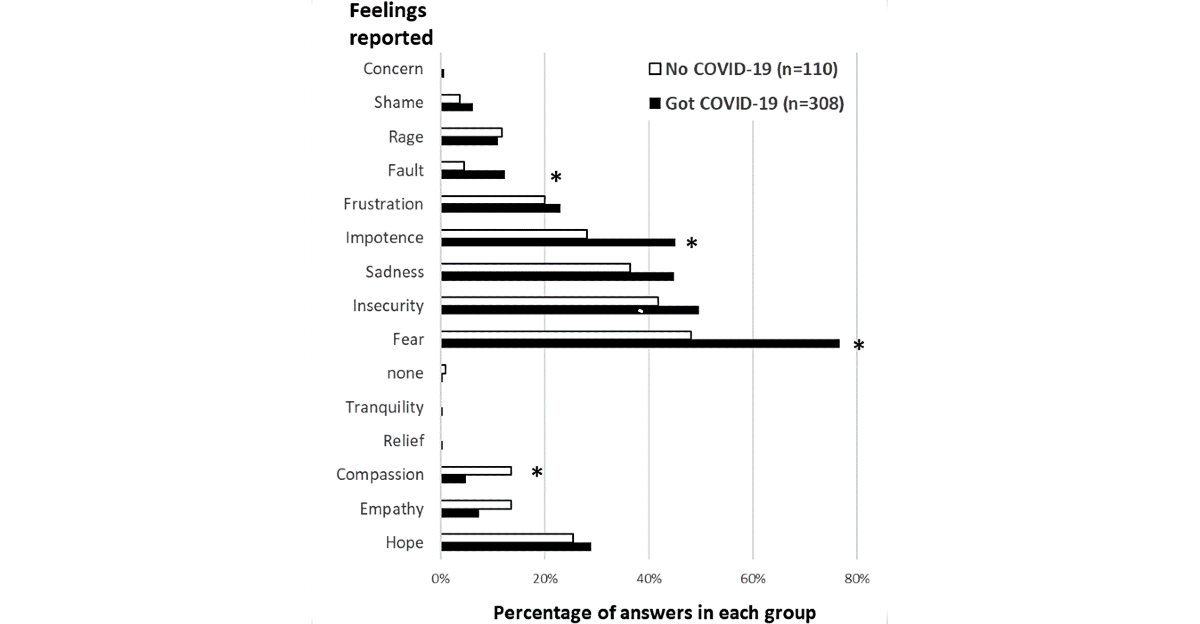
Feelings reported by the health professionals in the survey, showing frequencies of answers in the group reporting experience of acquiring COVID-19 (black bars) in comparison with those that did not acquire COVID-19 (white bars). Asterisks indicate significant differences (*P*<.05) between the two groups, as indicated by chi-square analysis.

We also asked that the participants that had COVID-19 to report their feelings during the pandemic period by using not only closed options but also allowing an additional open choice. Figure 1 shows the word cloud images of positive (Figure 1A) and negative (Figure 1B) feelings reported by all the health professionals responding to the COVID-19 perception survey, highlighting the hope and fear of COVID-19 as the predominant feelings, respectively. In this question, 110 participants who did not get COVID-19 chose to answer, thus allowing a quantitative analysis comparing both groups of respondents—those who got COVID-19 and those who did not—confirming that the 3 most recurrently stressful/negative feelings described by those who got COVID-19 were fear of COVID-19 (243/315, 77.0%), insecurity (158/315, 50.0%), and sadness (142/315, 45.0%), as shown in Figure 2. Positive feelings were also reported, including hope, empathy, compassion, relief, and tranquility (Figure 1A). The only significant difference between the 2 groups was found in the feeling of compassion, which was frequently more reported in the group that did not have COVID-19 (Figure 2). The group that got infected expressed some negative feelings at a higher frequency than those that did not get COVID-19, including fear (243/315, 77.1% vs 509/1061, 48.0%), impotence (142/315, 45.1% vs 297/1061 28.0%), and fault (38/315, 12.1% vs 567/1061, 53.4%), respectively. Insecurity, sadness, frustration, rage, shame, and concern were similarly reported. To assess the effect of the pandemic on stress, regardless of whether infected or not with SARS-CoV-2, we elaborated a question with affirmative sentences in which they could mark more than one option (Table 4). Approximately 43.9% (604/1376) of the health professionals pointed to “*I had an* anxiety due to COVID-19,” whereas 43.1% (593/1376) selected “*I experienced difficulties in falling asleep.*” Furthermore, 32.9% (453/1376) reported “*I had difficulty in concentrating*” and 28.0% (385/1376) reported “*I experienced traumatic situations at work*” (Table 4). From those who pointed *difficulties in falling asleep, in concentrating and lost interest in activities,* 60.0% (826/1376) also reported having a regular or bad institutional support. In addition, during the pandemic, 15.8% (217/1376) developed depression, 33.6% (463/1376) developed general anxiety, and 8.2% (113/1376) developed panic disorder (Table 4). According to our survey, to face the pandemic challenges and to deal with difficulties in this period, Brazilian health professionals received emotional support from family or friends, or from religion, spirituality, or faith (918/1376, 66.7%), and only 8.6% (119/1376) accessed professional psychological and teletherapy services (Table 4). A large percentage (1170/1376, 85.0%) reported receiving support from their immediate bosses at work, half of whom were considered as good/excellent and the other half as regular/bad (Table 4).

**Table 2:** Labor characteristics of the working places and personal protection equipment and COVID-19 information acquired by health professionals who participated in the national survey (N=1376).

Labor information			Respondents, n (%)
**Work during the pandemic**			
	Not working (unemployed)	164 (11.9)	
	Working on the front line of COVID-19	546 (39.7)	
	Working, not on the front line of COVID-19	566 (41.1)	
	Retired/temporarily away owing to comorbidities	100 (7.3)	
**Health system working place**			
	Public sector	636 (46.2)	
	Private sector/philanthropy hospitals	323 (23.5)	
	Both public and private sectors	127 (9.2)	
	Family residences	71 (5.2)	
	Health education institute	18 (1.3)	
	Web-based surveillance	11 (0.8)	
	Not working (retired/unemployed, others)	164 (11.9)	
**Receive sufficient information to prevent infection**			
	Yes	996 (72.4)	
	No	360 (26.2)	
	Did not answer	20 (1.5)	
**Have access to adequate safety equipment at work**			
	Yes	1057 (76.8)	
	No	319 (23.2)	
**Considered as a person from the risk groups**			
	No	882 (64.1)	
	Yes	494 (35.9)	
**Perceived alterations in daily routines**			
	Yes	970 (70.5)	
	No	406 (29.5)	
**Sources of information about COVID-19**			
	Ministry of Health/World Health Organization websites	551 (40.0)	
	Television	368 (26.7)	
	Internet sites/Facebook/Instagram	254 (18.5)	
	Newspapers and journals	109 (7.9)	
	At work	10 (0.7)	
	Radio	12 (0,9)	
	Refuse to get more information	6 (0.4)	
	Friends and family members	20 (1.5)	
	Science journals	23 (1.7)	
	Other media/all the sources	23 (1.7)	

**Table 3:** COVID-19 pandemic impact on Brazilian health professionals who participated in the national survey.

COVID-19 self-reported information		Respondents, n (%)
**SARS-CoV-2 infection**		
	Survey participants	1376 (100.0)
	Got infected	315 (22.9)
	Did not get infected/do not know	1061 (77.1)
**Symptoms developed (n=315, among only responders with COVID-19)**		
	One or two symptoms	33 (10.5)
	Three or more symptoms	263 (83.5)
	Headache	274 (87.0)
	Body pain	231 (73.3)
	Tiredness	228 (72.4)
	Loss of taste and smell	208 (66.0)
	Dry cough	171 (54.3)
	Fever	152 (48.3)
	Diarrhea	144 (45.7)
	Breath difficulty	128 (40.6)
	Minor symptoms (chest pressure, skin eruptions, conjunctivitis, vomiting)	149 (47.3)
	Asymptomatic	17 (5.4)
**Worsening of the clinical symptoms (n=315, among only responders with COVID-19)**		
	No worsening	287 (91.1)
	Hospitalized in the infirmary	19 (6.0)
	Hospitalized in the intensive care unit without intubation	8 (2.5)
	Hospitalized in the intensive care unit with intubation	1 (0.3)
**Where he/she presumes to have got infected (among only responders with COVID-19)**		
	Positive history of COVID-19	315 (100.0)
	Working in a health facility	193 (61.3)
	Do not know	48 (15.2)
	From family or friends	42 (13.3)
	Public transportation	16 (5.1)
	Supermarket/others	16 (5.1)
**Persons living in the same place got COVID-19 (n=315, among only responders with COVID-19)**		
	Yes	152 (48.3)
	No	134 (42.5)
	Do not know	29 (9.2)
**Persons living in the same place got COVID-19 (n=1061, among responders with negative history of COVID-19)**		
	Yes	91 (8.6)
	No	816 (76.9)
	Do not know	154 (14.5)
**Think have transmitted it to family/friends (n=315, among only responders with COVID-19)**		
	Yes	85 (27.0)
	No	181 (57.5)
	Do not know	49 (15.6)

**Table 4:** Effect of the COVID-19 pandemic on mental health/stress situations of Brazilian health professionals who participated in the survey (N=1376).

Answers concerning stress at work		Respondents, n (%)
**Agreement with this statement**		
	I had an anxiety due to COVID-19	604 (43.9)
	I experienced difficulties falling asleep	593 (43.1)
	I had difficulty concentrating	453 (32.9)
	I lost interest in activities I used to do	447 (32.5)
	I experienced traumatic situations at work	385 (28.0)
	I do not feel safe leaving home	372 (27.2)
	I did not go through these issues	274 (19.9)
	I had the need to seek psychological help	182 (13.2)
**Have family people depending on special care**		
	Yes	662 (48.1)
	No	714 (51.9)
**Diagnosis of adjustment disorder during pandemic**		
	No	612 (44.5)
	Yes, general anxiety	463 (33.6)
	Yes, depression	217 (15.8)
	Yes, panic	113 (8.2)
**Received emotional support from others**		
	Yes, from friends/family/religion/social networking	918 (66.7)
	Yes, from professional support	119 (8.6)
	No	339 (24.6)
**Received support from immediate boss at work**		
	Excellent	202 (14.7)
	Good	387 (28.1)
	Regular	306 (22.2)
	Bad	269 (29.5)
	Do not have bosses	212 (15.4)

**Figure 3:**
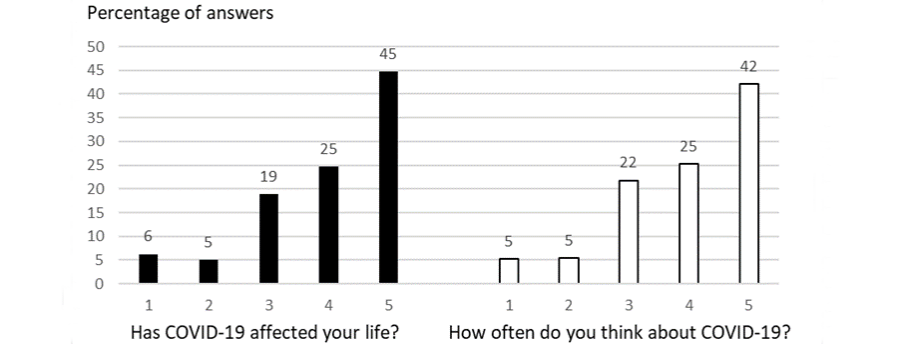
Scores attributed by the health professionals participating in the survey to 2 questions regarding general perception of the impact of the COVID-19 pandemic in their life. Score 1 represents none/few and score 5 represents very much/very often/a lot. The questions were “Has COVID-19 affected your life?” (answers in black bars) and “How often, since the beginning of the pandemic, do you think about COVID-19?” (answers in white bars).

The survey ended with 2 questions asking for a general opinion based on a 5-point scale and related to the general impact of COVID-19 in their lives (Figure 3). Question A: *Has COVID-19 affected your life?* (1=did not affect and 5=affected very much) and question B: *How often, since the beginning of the pandemic, do you think about COVID-19?* (1=almost never, 5=very often). Most of the respondents (963/1376, 70.0%) felt affected (344/1376, 25.0%) or very affected (619/1376, 45.0%) by the COVID-19 pandemic. This was in accordance with their answer to the second question, where a high proportion of health sector workers thought a lot (344/1376, 25.0%) or very much (578/1376, 42.0%) about the disease. Considering that, the higher the proportion of health sector workers overanalyzed about the disease, the greater the anxiety of the individual regarding COVID-19 was expected—both questions indicate this scenario of anxiety due to COVID-19 among most of the health care professionals.

## Discussion

### Principal Findings

At the end of 2019, COVID-19 was described as a disease that could be easily transmitted and rapidly spread by the SARS-CoV-2 virus [1,31]. Therefore, health professionals are one of the most exposed groups to this disease and to its psychosocial consequences as they are responsible for caring and dealing with patients infected by the virus on a daily basis [32,33]. The nation-by-nation number of deaths and infections of health professionals is still increasing [34,35]; in September 2020, about 570,000 health professionals became infected with the SARS-CoV-2 virus and 2500 died due to the disease in the Americas [28].

Our purpose in this study was to identify the impact of the COVID-19 pandemic on the life and work routine of Brazilian health professionals through the analysis of their perceptions and feelings during this period. Most health professionals who responded to the survey were females, European-derived, aged 31-40 years, and located in the southeast of Brazil, the most populous region. The data from our research showed a higher percentage of female health professionals’ participation in relation to males (our data: 1159/1376, 84.2% women and 215/1376, 15.6% men) confirming the literature, which shows that a greater number of health professionals in Brazil are females [32,33] and females are the most effective at work in the face of the COVID-19 pandemic [36]. Interestingly, we verified through our results similar impressions reported by individuals of both genders. Among the health professionals who worked during the pandemic, women represented the largest proportion—they also being the ones that perform the major care functions at home. Even in different studies carried out in Latin American countries, women constituted the highest proportion: Chile (72.6%) [37], Ecuador (68.3%) [38], Argentina (71%) [39], Bolivia (72.9%) [40], and Peru (71%) [41]. According to the United Nations Entity for Gender Equality and the Empowerment of Women [36], 70% of the global health workers are women (eg, nurses, midwives, community health workers) also working as cleaners, caterers, and launderers in health facilities; they have few leadership positions (30%) and lower salaries. In the pandemic scenario, women have been in a huge demand and have been professionally highly affected as children and the older adults depend on them even more, without schools or helpers to support them [42].

According to our survey, despite the highly stressful scenario, these female health workers did not respond differently from men in this pandemic situation, especially in terms of their feelings. They also felt the fear of COVID-19, sadness, hope, empathy, and insecurity while they cared for their family’s demands and social and economic problems. Literature shows that women are dealing with these health and stressful issues and social and economic problems totally by themselves, thus highlighting the need for creating gender-specific programs to help these women in the near future [36,42]. Some authors such as Campos et al [43] reported that even though most health care professionals are females, the death risk is higher (52.8 times higher) among younger men than among older women. They justified the higher death rates among younger men to be caused by the highly patriarchal nature of the Brazilian society with a very strong masculine pride and that men do not acknowledge their fragility or seek for assistance.

During the pandemic, the problems in the health care environment included the use of PPE that was intended for other individuals (eg, size for bigger men used by women or smaller persons) and even the absence of these materials as well as life support to use with the patients (eg, respiratory equipment). As the pandemic spread across the globe, the adequate provision of PPE for health professionals was a constant concern [44]. A cross-sectional study conducted in Latin America (Brazil, Colombia, and Ecuador) showed that at least 70% of the health professionals reported a lack of PPE [45]. This concern was reinforced by the answers of our participants, in which 1 in 5 complained about missing PPE such as face shields, masks, and laboratory coats. It is important to notice that the distribution of PPE to health institutions should be a government policy, especially in Brazil that has a huge public system called the Sistema Único de Saúde [46] that is always in massive demand and that requires mobilization of the national health industry to respond to the challenge of facing the pandemic. Unfortunately, this has not been done and the costs of PPE have been increased [47]. The scarcity of PPEs has also been reported in other Brazilian studies with smaller groups and in other countries [41,43,44,48], especially in those needed to protect frontline health professionals. In Italy, PPE shortages might be among the relevant factors contributing to the high burden of infection and hospital staff deaths, similar to what our survey indicated for Brazilian health workers [49].

Based on the fact that the recent vaccines developed against coronavirus are still not available for everyone in all countries, including Brazil [50], and that the number of new infections is growing at an alarming rate, especially those caused by new mutant strains [51], the knowledge about preventive steps is still essential to disrupt the chain of virus transmission among health professionals. In our study, the astonishing evidence was that 1 in 4 health professionals (26%) indicated a lack of enough information to protect themselves from COVID-19. It means that these health professionals work with insecurities and worry about being infected during their journey times—many of them who work as frontline health care professionals. Some studies described different aspects of health professionals from Brazil during the pandemic with lesser numbers of participants from specific states or regions and different evaluation aspects, sometimes including examining the psychological impact of the COVID-19 pandemic such as those reported by Campos et al [43], Duarte et al [52], and Cotrin et al [53]. Some of these studies showed that Brazil had the largest preponderance of death records caused by COVID-19, especially among nursing professionals, because of several factors such as direct contact with patients, the frequency in performing different procedures, and the lack or inadequate use of PPEs, among others. Our work added to these factors that misinformation (61%) contributed to the lack of precise knowledge about COVID-19 since almost 1 in 5 workers choose social networks as their source of information. The profusion of news on social networks, most of them without any validation on their authenticity, is becoming a huge social problem that compromises the ability to distinguish between facts, opinions, or fake news [54]. The problem is so serious that Dr Tedros Adhanom Ghebreyesus, the WHO Director-General called this news situation as an infodemic that should be fought against, leading to some efforts to create strategies to help on this issue [22,55]. The need for further awareness campaigns and knowledge of safe interventions to combat the spread of COVID-19 still remain, requiring that the health sectors increase the access to precise information about this disease [56]. These data also reinforced the identification of these workplaces as high-risk environments in Brazil as well as in other countries [57-59]. Although some studies with smaller groups pointed that economic income and educational qualification had some correlation with COVID-19, we did not observe them as a direct factor to be considered in this group. It is important to notice that our group showed a professional distribution similar to that described by national and international reports of the Brazilian medical team, which may suggest that these factors as more related to specific groups or regions in our country [53].

From the beginning of the SARS-CoV-2 outbreak, concerns have been raised about its effect on mental health [60,61]. According to WHO, mental health is defined as “*a state of well-being in which each individual realizes their own potential and can cope with the normal stress of life, can work productively and is able to contribute to their community,*” and it is more important than physical health, especially when it comes to stressful situations such as the COVID-19 pandemic [62]. Several studies have been published describing the mental profile of patients with COVID-19 who developed symptoms of anxiety, depression, psychological distress, and insomnia [63-65]. An American survey included 1651 respondents from all 50 states and reported that 60% of the health professionals had a higher risk of emotional distress/burnout during the COVID-19 pandemic [66]. Hair cortisol evaluation is a suitable biomarker for an individual’s exposure to stressful events. A study conducted in Argentina on 234 health professionals showed that 40% of the sample population presented hair cortisol values outside of the healthy reference range in the course of the COVID-19 pandemic, thereby showing a direct correlation with the perceived stress and the emotional exhaustion component of burnout [39]. In Canada, by surveying health professionals, Wilbiks et al [67] described that there was an elevated level of depressive symptomatology in that population. The prevalence of stress, anxiety, and depression in frontline health care professionals caring for patients with COVID-19 was already described for some small groups such as those described with a convenience sample of 364 health workers, including physicians, nurses, pharmacists, and laboratory technicians [58]. Like our study that identified positive feelings from the participants, they described positive attitudes from all participants with mostly moderate COVID-19 psychological stress levels.

The literature also described a systematic review that evaluated 29 studies, with a total sample size of around 22,000 health professionals. Similarly to our survey in which several health professionals experienced anxiety due to COVID-19 (N=9680, 44%), depression (N=7480, 34%), and insomnia (N=7260, 33%), the review showed that 21 papers described the prevalence of depression, 23 reported the prevalence of anxiety, and 9 studies have reported the prevalence of stress [68]. COVID-19 changed the lives of everybody worldwide [69], and our survey reinforced that the Brazilian health professionals were also affected at a high level at 70% (N=963) and this is apparently directly associated with higher levels of psychological and physical stress.

### Limitations

Our study has some limitations, which need to be considered. The findings are not generalizable to all categories of health care professionals, as it is a compilation of all respondents’ impressions. Another important piece of information that should be deemed is the total period of the COVID-19 pandemic. We evaluated the perceptions and feelings of these professionals in a specific time frame. Therefore, longitudinal studies are recommended in Brazil. Despite the self-report questionnaire being one of the most widely used assessments, its use rather than a clinical assessment reduced the power of our findings. Another limitation of this study was that most of the respondents were those who used or operated the internet, which only constitutes a partial section of the society. However, this study could suggest a general overview of the perceptions and feelings present in the health professionals in Brazil.

### Conclusions

In every country, during the COVID-19 pandemic, health care professionals had to work under pressure with risks of affecting their physical and mental health, by being on the front line and assisting to save lives. Our data showed that the COVID-19 pandemic affected overall 70% of the Brazilian health professionals, according to their answers to our survey. However, most of the feelings did not change when comparing those who did get infected to those who did not, men or women, suggesting that to be exposed to this work environment and the pandemic situation are enough to develop negative feelings, such as fear of COVID-19, sadness, and insecurity, despite keeping “alive” their hope. These negative feelings are probably maintained by knowing situations such as (1) absence of Brazilian strategies at the national level for mass testing of the population, (2) absence of effective public policies that reduce the cases of COVID-19, and (3) absence of sanitary measures carried out in a centralized manner by states and municipalities (not only guaranteed by calling the judiciary), especially in states where the epidemic is most severe. Altogether, these feelings and perceptions reported in this work are alarming and must be well addressed with interventions that enhance the quality of life of the health professionals. There is an urgent need for regular monitoring of potential stress disorders, aiming to reduce the associated side effects in the longer run. Therefore, health policymakers should plan actions to control and prevent mental disorders in this category of professionals as soon as possible. One of the actions that should be implemented in each hospital, clinic, and asylum is the creation of multidisciplinary groups that may attend and monitor the medical staff, including all involved, not only for training but also to dialogue and identify burnout situations before they deeply and irreversibly affect this group that is so stressed out in this pandemic. This also includes the assurance of vaccination (2 doses taken) for all of them.

## Abbreviations

PPE: personal protective equipment
WHO: World Health Organization
